# A recipe for dyadic collective intelligence for well-structured tasks: mix equal parts cognitive ability and confidence plus a pinch of social sensitivity

**DOI:** 10.1186/s41235-025-00655-0

**Published:** 2025-09-24

**Authors:** Matthew D. Blanchard, Eugene Aidman, Lazar Stankov, Sabina Kleitman

**Affiliations:** 1 School of Psychology (A18, Room 440), The University of Sydney, Sydney, NSW 2006 Australia; 2 School of Biomedical Sciences and Pharmacy, University of Newcastle, Callaghan, NSW Australia; 3 Defence Science and Technology Group, Human and Decision Sciences Division, Third Avenue, Edinburgh, SA 5111 Australia

**Keywords:** Collective intelligence, Two heads better than one, Confidence, Dyads, Teams, Latent profile analysis

## Abstract

A collective intelligence factor (CI) was introduced by prior research to characterise the cognitive ability of groups. Surprisingly, individual intelligence did not predict CI. Instead, it correlated with individual social sensitivity, the equality of conversational turn-taking, and the proportion of females in a group. However, these findings may depend on the type of tasks completed by groups. Our study re-examined these relationships by (1) testing the robustness of the CI factor in dyads using well-structured tasks guided by the Cattell–Horn–Carroll (CHC) model of intelligence; (2) exploring the relationship between dyadic CI and metacognitive confidence, which is known to influence group processes and outcomes; and (3) identifying the psychological characteristics of distinct dyad types using latent profile analysis. We measured CI in 105 undergraduate dyads using three group tasks aligned with the broad abilities of the CHC model. Individual intelligence was assessed using Raven’s Advanced Progressive Matrices. We also measured social sensitivity, proportion of females, equality of turn-taking, working memory, and personality. Results indicated that individual intelligence and confidence were the strongest predictors of dyadic CI for well-structured tasks, contrasting with previous findings emphasising social factors. While we replicated the relationship with social sensitivity, we did not replicate the findings for equality of turn-taking or gender composition. Latent profile analysis identified three psychological profiles: dyads performing consistently high individually and collectively, those performing consistently low, and those performing better collectively than individually. Our “smarter” dyads consisted of intelligent and confident individuals with higher social sensitivity. These findings suggest that, in dyads performing well-structured tasks, individual cognitive abilities and confidence play significant roles in CI. This challenges the emphasis on social factors and underscores the importance of task selection.

**Supplementary Information:**

The online version contains supplementary material available at 10.1186/s41235-025-00655-0.

## Significance Statement

Dyads are commonly utilised to make decisions because there is a belief that “*two heads are better than one*”. However, not all dyads outperform individuals, and the factors contributing to their success remain inadequately understood. This knowledge gap limits our ability to systematically develop effective collaboration, which is essential for improving real-world dyadic outcomes. Our findings address this gap by extending collective intelligence (CI) to dyads completing well-structured tasks. Our findings challenge prior research by showing that individual intelligence, coupled with confidence and social sensitivity, plays a more important role in dyadic CI for well-structured tasks than previously recognised. By employing a novel battery of tasks guided by the Cattell–Horn–Carroll model of intelligence and latent profile analysis, we identified three distinct types of dyads: those who consistently performed at a high level; those who consistently performed at a low level; and those who performed better collectively than individually. This last profile enhanced performance through strategic dominance by allowing the more competent member to dominate discussions and decision choices. This challenges prior research that claimed equality of conversational turn-taking was associated with higher CI. Our research refines the theoretical framework of CI by offering a more precise “recipe” for successful dyadic collaboration in well-structured tasks. Our findings can inform dyad selection practices, by emphasising individual intelligence, confidence, and social sensitivity, and guide training programmes aimed at helping members identify and leverage the strengths of the more competent member. These insights have practical implications that can improve dyadic performance in real-world settings.

**Supplementary Information:**

The online version contains supplementary material available at 10.1186/s41235-025-00655-0.

## Introduction

Collective decision-making is a complex process influenced by human interaction, cognitive ability, and metacognitive confidence (e.g. Bahrami et al., 2010; Hill, 1982; Hinsz, 1990; Zarnoth & Sniezek, 1997). Woolley et al. (2010) introduced the concept of *collective intelligence* (CI) as a group’s ability to perform across a wide variety of tasks, emphasising nuanced factors like one’s ability to perceive others’ emotions and interaction processes, rather than cognitive ability. This seminal work provided a new perspective for understanding why some groups thrive when making collective decisions and others do not. However, subsequent research (e.g. Graf-Drasch et al., 2022; Rowe et al., 2021) suggests Woolley et al.’s findings may be task dependent and limited in generalisability, particularly for well-structured tasks which have a single, objective solution.

To address these limitations, our study re-examined CI by focusing on dyads and employing well-structured tasks guided by the Cattell–Horn–Carroll (CHC) model of intelligence. This approach allowed for a more precise assessment of the relationship between individual intelligence and dyadic CI. Additionally, we explored the role of metacognitive confidence, given its central influence on group decision-making outcomes (e.g. Bahrami et al., 2010; Kerr & Tindale, 2004; Koriat, 2015), and how it may uniquely contribute to CI.

Our primary aim was to clarify the relationship between individual intelligence and CI for dyads on well-structured tasks. We hypothesised that individual intelligence would positively predict dyadic CI, challenging previous findings that social factors are more critical than intelligence. Furthermore, we investigated the relationship between metacognitive confidence and CI to understand how individuals’ confidence levels impact collective performance. Finally, we applied latent profile analysis (LPA) as a novel, person-centred approach to identify distinct psychological profiles of dyads based on their individual and dyadic scores on intelligence, metacognitive confidence, and bias (the accuracy of confidence). This methodology allowed us to differentiate dyads by their performance patterns, revealing common changes in outcomes from individual to dyadic performance and offering insights into optimising group compositions for enhanced CI.

### Collective intelligence

CI broadly refers to the common finding that groups tend to perform better than individuals (e.g. Blanchard et al., 2023, 2024; Gordon, 1924; Hill, 1982; Kameda et al., 2022; Kerr & Tindale, 2004; Kurvers et al., 2016; Williams & Sternberg, 1988). Various conceptualisations of CI exist, including consensus-seeking groups and the wisdom-of-crowds. The present study focused on Woolley et al.’s (2010) definition of CI for consensus-seeking groups as “the general ability of the group to perform a wide variety of tasks” (p. 687), rooted in the idea that CI parallels individual intelligence but at the group level.

Woolley et al. (2010) modelled CI on Spearman’s theory of general intelligence for individuals (Spearman, 1904), using a battery of tasks selected based on the McGrath Group Task Circumplex (McGrath, 1984). This is a taxonomy categorising tasks by the coordination processes required for completion. Woolley and colleagues selected at least one task from each category (generation, decision-making, negotiation, and execution) to provide a broad assessment of group ability. In the first study, their tasks included brainstorming possible uses for a brick, Raven’s Advanced Progressive Matrices (RAPM), moral reasoning, negotiating plans for a shopping trip, and typing a difficult text under time constraints. A factor analysis across these tasks yielded a first-order CI factor (“c”), analogous to Spearman’s “g” for individual general intelligence, suggesting that CI could predict group performance across varied contexts.

#### Original research findings

In two studies involving 192 groups of three to five members, Woolley et al. (2010) found that CI accounted for approximately 43–44% of the variance in group performance across tasks. CI also predicted performance on a criterion task (study 1: computerised checkers, *r* = 0.52; study 2: architectural design, *r* = 0.28). Their results linked higher CI scores with greater social sensitivity, equality of turn-taking (i.e. lower variance in speaking turns), and a higher proportion of female group members, although this last finding was fully mediated by social sensitivity. These findings have been replicated several times by Woolley and colleagues (Aggarwal et al., 2019; Engel et al., 2014, 2015; Kim et al., 2017; Riedl et al., 2021), suggesting that CI is distinct from individual intelligence, with a weak correlation between average individual intelligence and CI (*r* = 0.15).

These studies make two major claims. First, social factors, such as accurately perceiving others’ emotions (social sensitivity) and sharing conversational turns equally, are more important for group performance than individual intelligence. However, the finding for social sensitivity requires clarification, as the instrument used for its measurement, Reading the Mind in the Eyes test, has limitations. Research suggests it often produces a ceiling effect in neurotypical populations so it may not be effective at assessing individual differences (Black, 2017), often has low internal consistency (e.g. Harkness et al., 2009; Ragsdale & Foley, 2011), and is a mixed measure of emotion perception, vocabulary knowledge, cognitive empathy, and affective empathy (Kittel et al., 2022; Olderbak et al., 2015). This finding requires replication with a different tool that assesses emotion perception. The second major claim is that groups possess general abilities and characteristics that allow them to perform well across a wide range of contexts. This challenges the long-held belief that groups require specialised abilities and skillsets (e.g. expertise) to perform effectively in specific contexts (Cohen & Bailey, 1997; Hollingshead & Poole, 2012; Steiner, 1972).

Woolley et al.’s studies present CI as a robust construct, independent of individual intelligence, that predicts group performance across various contexts. However, the claim that CI “has been well established in the literature” (Askay et al., 2019, p. 492) is contradicted by mixed results from independent research.

#### Independent research and meta-analytic findings

Credé and Howardson (2017) reanalysed the data from 6 studies, finding that CI accounted for limited variance in group performance, and the CI tasks had low internal consistency. Barlow and Dennis (2016) failed to replicate a dominant CI factor for virtual, text-based groups, and social sensitivity showed inconsistent relationships with performance on each of the group tasks. Bates and Gupta (2017) observed that individual intelligence explained much of CI’s variance, while Woolley’s key predictors (social sensitivity, equality of turn-taking, and proportion of women) showed no significant relationship with CI. Guided by Horn and Cattell’s (1966) Gf-Gc theory of individual intelligence, Rowe et al. (2024) used group tasks designed to assess individual intelligence and found that two factors, rather than one, best accounted for variance in CI. Neither social sensitivity nor equality of turn-taking was related to the CI factors.

A meta-analysis by Graf et al. (2019) proposed a 3-factor structure for CI (idea generation, conflict resolution, and task execution) which challenged Woolley’s 1-factor model and aligns with broader team research. For example, LePine et al. (2008), in a meta-analysis of 138 studies, found a second-order model with three broad processes (transition, action, and interpersonal) and a higher-order teamwork process factor that was correlated with team performance. In a review, Hackman and Morris (1975) also showed that group interaction processes strongly related to group performance. These findings suggest that Woolley’s CI captures teamwork processes and dynamics rather than a cognitive construct analogous to individual intelligence.

Rowe et al. (2021) provided additional insights into CI’s limitations in a meta-analysis, finding a weak correlation with average individual intelligence (*r* = − 0.05 to 0.34). Tasks such as moral decision-making, negotiating a shopping trip, and collaboratively typing are not typical intelligence measures, potentially limiting CIs relevance as a cognitive construct. These inconsistencies raise questions about CI’s robustness and its dependence on the types of tasks chosen.

Laughlin (2011) distinguished between intellective and judgmental tasks, illustrating how task structure influences group decision-making. Building on Laughlin’s work, Graf-Drasch et al. (2022) categorised tasks used in prior CI research as either well-structured or ill-structured and examined CI for each type. Woolley’s CI emerged only for well-structured tasks, which have a clear strategy leading to a single correct solution, unlike ill-structured tasks that are ambiguous, allow multiple solutions, and emphasise group interaction. These findings imply that Woolley’s CI may only apply to well-structured tasks, and its relationship with individual intelligence remains unclear. They also raise two critical questions: Could individual intelligence predict dyadic CI when measured using well-structured tasks? Prior research suggests this is likely, given that individual intelligence tends to relate to group performance on well-structured tasks (Bruine de Bruin et al., 2007, 2012, 2019; Del Missier et al., 2012). Furthermore, extensive research demonstrates the central role of individual intelligence in group performance (Barrick et al., 1998; Bell, 2007; Devine & Philips, 2001; Imbimbo et al., 2020; LePine, 2003, 2005; LePine et al., 1997; Stewart, 2006). Second, are Woolley’s key predictors (social sensitivity, equality of turn-taking, and the proportion of females) related to dyadic CI for well-structured tasks? Inferring others’ emotions is crucial for effective social interactions (Lopes et al., 2003), and equality of turn-taking is associated with higher decision quality (Janis & Mann, 1977; Vroom & Yetton, 1973). Gender differences in communication styles, with females tending to be more supportive and males more dominant (Anderson & Leaper, 1998; Carli, 2001; Carli & Bukatko, 2000; Fishman, 1978; Leet-Pellegrini, 1980), may be more pronounced for larger groups (3–5 members) and ill-structured tasks, where more communication is generally required. Thus, for dyads, the cognitive requirements of well-structured tasks may drive gender differences. For example, there may be a small male advantage for tasks that primarily require Fluid Reasoning (e.g. Halpern et al., 2007), and there may be a small female advantage for tasks that primarily require verbal abilities (e.g. Reilly et al., 2019).

#### Methodological considerations

Replicating CI with varied methods is crucial for assessing its robustness (Botvinik-Nezer et al., 2020). Woolley’s reliance on theoretical taxonomies, like the McGrath’s Group Task Circumplex, often lacks empirical support, leading to overlapping task categories that can blur construct clarity (Devine, 2002). These taxonomies often fail to distinguish between the characteristics that drive team effectiveness for different types of teams (e.g. teams engaged in building a house versus those engaged in conducting scientific research; Cohen & Bailey, 1997). Theoretical taxonomies typically sample items based on their subjective correspondence with perceived group characteristics (e.g. generate, choose, negotiate, and execute). This approach is guided by theory but lacks empirical validation. Bell’s (2007) meta-analysis found that group task taxonomies had no moderating effect on the relationship between individual intelligence and group performance. Most studies of Woolley et al.’s (2010) CI used the McGrath Group Task Circumplex to guide the selection of group tasks. Suggesting that their selection of items may not adequately cover the hypothesised construct to capture the latent properties of the cognitive processes that drive group performance.

In contrast, the CHC model of intelligence offers an empirically validated framework for understanding the cognitive processes involved with individual performance on a wide variety of tasks (e.g. Carroll, 1993; Horn & Cattell, 1966; McGrew, 2009). The CHC model synthesises over a century of research and encompasses 16 broad abilities, such as Fluid Reasoning (i.e. abstract reasoning that has little dependence on acquired knowledge), Crystallised intelligence (i.e. acquired knowledge that is culturally relevant), and Quantitative Knowledge (i.e. acquired knowledge about mathematics). Each of these abilities is supported by extensive psychometric evidence and has been linked to performance on a wide range of tasks.

By using the CHC model to guide the selection of well-structured tasks, we can reliably assess the relationship between individual intelligence and dyadic CI. This approach allowed us to examine the cognitive abilities that are most relevant to CI. For example, Fluid Reasoning may help us understand how dyads solve novel problems, while Crystallised Intelligence can shed light on how shared knowledge and unique knowledge contributes to group decisions.

However, it is important to note that the CHC model is designed to explain cognitive abilities at the individual level and does not account for the interaction processes that emerge when people work together in groups. Interaction processes (e.g. communication patterns, coordination, and conflict resolution) play a critical role in group performance (Hackman & Morris, 1975; Mesmer-Magnus & DeChurch, 2009). These processes can lead to synergistic effects where the group’s performance exceeds the sum of its parts (e.g. high CI), or conversely, to process losses where group performance is harmed (Steiner, 1972).

While the CHC model provides a strong foundation for assessing the cognitive components of dyadic performance, it was not designed to capture these dynamic social interactions. Incorporating measures of interaction processes could enhance our understanding of CI by revealing how cognitive abilities, interaction processes, and social dynamics jointly contribute to group outcomes. However, integrating these factors requires a more complex research design and is beyond the scope of the present study. Our focus is on isolating the impact of individual intelligence on dyadic CI using well-structured tasks guided by the CHC model.

The hierarchical structure of the CHC model, which includes both broad and narrow cognitive abilities, provides a nuanced framework for analysing the interplay between individual intelligence and CI. This approach addresses previous methodological limitations by grounding our task selection in a validated theoretical model.

#### The current research

Addressing previous limitations in CI research, the primary aim of our study was to examine two critical questions: First, does individual intelligence predict dyadic CI when measured using well-structured tasks? We used parallel forms of a Fluid Reasoning test to assess both individual intelligence and CI, along with additional measures of Crystallised Intelligence and Quantitative Knowledge to measure CI. This approach allowed for a more precise investigation of the relationship between intelligence at the individual and collective levels. Prior research outside the CI paradigm indicates a moderate correlation between individual cognitive ability and group performance for these tasks (Del Missier et al., 2012; Bruine de Bruin, 2019).

*H1*: We hypothesised that individual intelligence would positively predict CI for well-structured tasks, after accounting for the other key variables.

Second, do social sensitivity, equality of turn-taking, and proportion of females, identified by Woolley et al. (2010), predict dyadic CI for well-structured tasks? We measured CI using tasks that capture Fluid Reasoning, Crystallised Intelligence, and Quantitative Knowledge. Two of these tend to show small male performance advantages; thus, CI may be greater for male than female dyads.

*H2a*: We hypothesised that the proportion of females would negatively predict CI, after accounting for the other key variables.

This conflicts with Woolley et al.’s finding that social sensitivity fully mediated the positive relationship between proportion of females and CI. We expected that the cognitive abilities required for well-structured task success would have a stronger influence on the relationship between gender composition and dyadic CI than an indirect effect through social sensitivity.

While social sensitivity and equality of turn-taking are associated with high-quality decision-making (Janis & Mann, 1977; Vroom & Yetton, 1973), independent CI researchers have failed to replicate Woolley and colleagues’ original results (Barlow & Dennis, 2016; Bates & Gupta, 2017; Rowe et al., 2024). Therefore, we did not expect these predictors to be significant after accounting for the other variables in the model.

*H2b*: We hypothesised that social sensitivity and equality of turn-taking would not predict CI, after accounting for the other key variables.

### Confidence and group decision-making

Research shows that confidence significantly influences both the processes and outcomes of group decisions. Sniezek and Henry (1989) found that groups tend to be more accurate and confident than individuals. Similarly, Kerr and Tindale (2004) observed that individuals with higher confidence often dominate discussions, exerting greater influence on the group’s final decision compared to less confident members. Bahrami et al. (2010) demonstrated that the “two heads are better than one” effect occurred when group members shared their confidence accurately. Blanchard et al. (2020) extended this by showing that overconfident dyads increased in decision-making errors more than underconfident or well-calibrated dyads, highlighting the role of metacognitive bias in group decision-making. Bias, or the degree of over- or under-confidence in one’s judgments, reflects one’s ability to accurately monitor their own performance. These findings demonstrate that confidence is a critical factor in group interactions and decision-making outcomes, suggesting its potential relevance to CI. This relationship remains underexplored in prior research.

Confidence is sometimes overlooked due to its close relationship with accuracy. However, confidence reflects distinct psychological processes. Koriat (2024) found that confidence monitored the likelihood of response replicability rather than accuracy. High confidence indicated that an individual would likely choose the same response if faced with the same question again, even if it was incorrect. This has implications for groups: high-confidence groups are likely to show consistency over time with similar tasks, whereas low-confidence groups may behave inconsistently. It is our belief that measuring both accuracy and confidence is essential for comprehensive group research.

In the present study, we extended previous findings by exploring the distinct roles of individual confidence and intelligence in predicting CI. Our second aim was to examine the relationship between individual confidence and dyadic CI.

*H3*: We hypothesised that individual confidence would positively predict CI, after accounting for other key variables.

Based on Koriat’s (2024) work, our third aim was to compare the predictors of collective confidence with those of CI to demonstrate that it captures unique information about dyads beyond CI alone. We expected that the relationships between collective confidence and the predictors would differ from those between CI and the same predictors.

*H4*: We hypothesised that collective confidence would have a distinct pattern of relationships with individual intelligence, individual confidence, and the other key variables, compared to CI.

### Profiling dyadic performance

The literature has predominantly used a variable-centred approach, focusing on average relationships between variables across the entire sample, to investigate CI. This provides valuable insights into general trends and correlations; however, it may overlook important differences between distinct types of groups. For example, Woolley et al. (2010) used an analytic approach that assumed the same relationships between variables applied equally to all groups, potentially hiding meaningful heterogeneity about how individual and group characteristics interact to influence CI.

To address this limitation and extend previous research, we adopted a person-centred approach by using LPA to identify clusters of dyads with similar psychological profiles across intelligence, confidence, and bias, measured at both the individual and collective levels. LPA allowed us to identify subgroups within the sample that shared unique behaviour patterns across these variables. This approach provides a more nuanced understanding of the different types of dyads. For example, LPA can reveal profiles of dyads that outperform their individual members (*two heads are better than one* effect) or dyads that collectively underperform compared to their members working alone. (*Two heads are worse than one* effect.) By including both individual and dyadic variables in the LPA model, we can capture shifts between individual and collective responses, thus addressing the heterogeneity of dyads providing valuable insights into possible distinct subgroups.

This method is a strategic departure that extends the approach of Woolley et al. (2010) because it simultaneously accounts for individual differences and group dynamics. It contributes to the theoretical framework of CI by providing a more detailed “recipe” of the ingredients that are associated with different types of dyadic performance. This information is essential for the formation of effective dyads and for developing interventions that enhance outcomes.

Our fourth aim was to identify psychological profiles of dyads using clustering based on intelligence, confidence, and bias. Given the exploratory nature of this analysis, we could not predict the exact number or nature of profiles that LPA would extract. However, we anticipated the emergence of multiple profiles, including at least one showing a *two heads are better than one* effect. Other possible profiles might reflect *two heads are the same as one effects (e.g.* high or low intelligence at both levels) and a *two heads are worse than one* effect. Furthermore, additional profiles could be shaped by variations in confidence and bias.

Our fifth aim was to examine differences between these distinct dyadic profiles on the key variables identified by Woolley and colleagues, as well as the individual difference variables described in the following section. This aim allowed us to explore how distinct dyadic profiles related to individual differences, potentially informing the selection of dyad members to improve dyadic performance or to prevent ineffective pairings.

### Individual differences

Working in a group is a complex task, and individual differences can influence group processes and outcomes. Key constructs include working memory, essential for holding information in mind for mental tasks (Baddeley, 1992) and Big Five personality, as meta-analyses show that Agreeableness, Conscientiousness, and Openness to Experience positively correlate with group performance (Bell & Kozlowski, 2002; Peeters et al., 2006). In this study, we examined the relationships between the LPA profiles and these individual difference variables.

### The present study

To investigate our hypotheses (presented in Table 1) and two exploratory aims, we employed well-structured tasks aligned with the CHC model. Individual intelligence and confidence were assessed using a Fluid Reasoning task, while CI and collective confidence were assessed using three tasks capturing Fluid Reasoning, Crystallised Intelligence, and Quantitative Knowledge. Following Woolley et al., (2010), we conducted a confirmatory factor analysis (CFA) to extract a single CI factor and a single collective confidence factor. We then fit two hierarchical regression models with the CI factor as an outcome variable in model 1 and the collective confidence factor as an outcome in model 2 and the following predictors: social sensitivity, equality of turn-taking, number of females, individual intelligence, individual confidence, and the individual differences variables.

**Table 1:** The hypotheses tested in the current study

Number	Hypothesis
H1	We hypothesised that individual intelligence would positively predict CI for well-structured tasks, after accounting for other key variables
H2a	We hypothesised that the proportion of females would negatively predict CI, after accounting for the other key variables
H2b	We hypothesised that social sensitivity and equality of turn-taking would not predict CI, after accounting for the other key variables
H3	We hypothesised that individual confidence would positively predict CI, after accounting for other key variables
H4	We hypothesised that collective confidence would have a distinct pattern of relationships with individual intelligence, individual confidence, and the other key variables, compared to CI

Given that individuals are nested within dyads, a multilevel model is typically recommended to account for interdependence within dyads (Gonzalez & Griffin, 2023; Kenny et al., 2006). Multilevel modelling requires variance at both the within-dyad and between-dyad levels for the outcome and predictors. In our study, however, CI was measured at the dyadic level through consensus-based responses, meaning there was no within-dyad variance in CI. Kenny and Kashy (2014; p. 591) note “if only a single outcome is obtained for each group, then group is treated as the unit of analysis, and special analytic methods are not required”. Therefore, in our case, the dyad is treated as the unit of analysis, and nesting individuals within dyads in a multilevel model is not appropriate.

We did not expect social sensitivity to have a significant positive relationship with CI. However, if Woolley et al.’s (2010) finding was replicated, we would conduct a mediation analysis to investigate the direct and indirect relationship between the proportion of females and CI, with social sensitivity as a mediator. We expected to find a negative direct relationship between the proportion of females and CI, contrasting with Woolley and colleagues, who reported that social sensitivity fully mediated a positive relationship.

We then fit an LPA model to identify clusters of dyads with similar psychological profiles across intelligence, confidence, and bias, measured at both the individual and collective levels. Finally, we conducted a series of between-subjects ANOVAs to test for differences between the identified profiles on Woolley et al.’s (2010) key variables (social sensitivity, equality of turn-taking, and proportion of females) and the individual differences measures.

## Method

### Participants

In return for partial course credit, 244 Australian undergraduate psychology students completed the study (158 females, 86 males, mean age = 20.82, SD = 4.12). Thirty-four participants were excluded from analyses who did not complete the protocol due to Qualtrics platform or computer crashes, timing constraints, or non-genuine attempts. Non-genuine attempts were identified through lab observations (where dyads did not engage as instructed), rapid response times suggesting random guessing, and self-reports from participants acknowledging non-genuine participation.

The final sample included 210 participants (133 females, 77 males, mean age = 20.79, SD = 4.33) who completed the study as 105 two-person groups. These dyads were formed as minimal groups. That is, participants were randomly allocated to dyads, and the majority (89%) did not know each other prior to participation in the study. However, 12 dyads indicated they had met before participation, with half meeting within the last 6 months. Given the small number of dyads that knew each other prior to the study and a non-significant correlation with CI (*r* = − 0.14, *p* = 0.15), we did not include familiarity in our analyses.

### Measures

#### CI tasks

For each of the following tasks except RAPM, items were presented in a fixed order and participants answered each question twice: first individually, then together with their teammate. This approach was employed so individual and collective decisions could be compared, enabling us to examine the two heads are better than one effect for each task. This repeated-measures approach is well established in dyadic decision-making research (e.g. Bahrami et al., 2010; Blanchard et al., 2020; Koriat, 2015).

*Applying decision rules* (ADR; Bruine de Bruin et al., 2007). This test included 10 items that each presented 5 DVD players, their prices, and ratings (very low to very high) on 4 attributes (i.e. picture quality, sound quality, programming options, and reliability of brand). Participants were provided with a fictive customer’s preferences on price and/or the 4 attributes and were required to select the DVD player that best matched their preferences. For example, participants were asked “*LaToya only wants a DVD player that has got a ‘Very High’ rating on Reliability of Brand. Which one of the presented DVD players would LaToya prefer?”* There was always one correct answer. Accuracy on this test is a mixed measure of Fluid Reasoning and Crystallised intelligence and possesses good internal consistency (0.73). After each item, participants provided a confidence rating ranging from 20% (guessing) to 100% (completely certain).

*Cognitive reflection test* (CRT; Frederick, 2005; Toplak et al., 2014). This test included 7 items composed of numerical problems that elicited biased judgments because people tend to rely on a heuristic instead of conducting a simple mental calculation. For example, participants were asked “*Together a bat and a ball cost $1.10. The bat costs $1 more than the ball. How much does the ball cost?”* Responses were free text. Accuracy on this test is a measure of Quantitative Knowledge (Otero et al., 2022) which is a broad cognitive ability and has demonstrated good reliability (0.72). After each item, participants provided a confidence rating ranging from 0% (guessing) to 100% (completely certain).

*Geography test* (Kleitman & Stankov, 2001). This test included 11 items that assessed participants’ knowledge of Australian geography. Each item presented a question with two response options, and participants were required to select the correct one. For example, participants were asked “*Which of the following states has a larger population: New South Wales or Victoria?*” Accuracy on this test is a measure of Crystallised Intelligence. After each item, participants provided a confidence rating ranging from 50% (guessing) to 100% (completely certain). The 11 items were selected from the 140 items used in the original study.

*Ravens advanced progressive matrices* (RAPM; Raven, 1938–1965). This test included 36 items. Each item displayed a 3 × 3 matrix of abstract figures that presented a horizontal and vertical pattern. The bottom right figure was blank, and participants were required to choose which of eight options completed the pattern. Accuracy on this test is a measure of Fluid Reasoning. Internal consistency on this test has been shown to be excellent for accuracy (0.80–81) and confidence (0.90–0.92; Blanchard et al., 2020, 2023). After each item, participants were asked to provide a confidence rating ranging from 12.5% (guessing) to 100% (completely certain) for the correctness of their response. The items from this test were split evenly to make 2 versions: individuals completed the 18 odd items, and dyads completed the 18 even items. This is known to produce equivalent short versions of RAPM.

#### Other measures

Each of the following measures was completed by individual participants.

*Composite Emotions Task* (Wilhelm et al., 2014). This 36-item test assessed social sensitivity or one’s ability to accurately perceive emotions. Each item presented a composite image composed of two photographs of the same face expressing different emotions (e.g. the top half of the face displayed anger, and the bottom half of the face displayed happiness). Each composite image presents two of six emotions: sadness, disgust, fear, happiness, anger, or surprise. Participants were directed to identify the emotion shown on either the top or bottom half of the face and responded by selecting which of the six emotions were displayed in the target half of the face. Accuracy on this test has been shown to possess excellent reliability (0.81).

*Medical Decision-Making Test* (MDMT; Jackson & Kleitman, 2014; Jackson et al., 2016, 2017). In this test, participants were told they were specialists in the Alpha virus, which can occur in multiple forms (regular or one of three mutations). They were given 3 min to memorise the pattern of associations between 9 symptoms and each form of the virus. For each of the 16 items, a fictive patient was presented with two symptoms. Participants diagnosed them as having the regular or one of the mutated forms of the virus. After each item, participants provided a confidence rating ranging from 25% (guessing) to 100% (completely certain) and decided whether to treat the patient immediately or request a blood test to provide further diagnostic information. Patients survived if treated following a correct diagnosis but died if treated following an incorrect diagnosis. Blood tests resulted in a correct diagnosis and treatment, but only 50% of untreated patients survived while waiting for blood test results. The goal was to save as many lives as possible. Accuracy on this test is a measure of short-term memory and Fluid Reasoning and has demonstrated good reliability (0.89–0.92). In the current study, this test was used to compute Bias scores: positive scores indicated overconfidence, negative scores indicated under-confidence, and scores approaching zero (± 10 percentage units) indicated unbiased confidence ratings (Keren, 1991; Stankov et al., 2014; Yates, 1990).

*Running letter span* (Broadway & Engle, 2010; Kane et al., 2004; Pollack et al., 1959). For each trial, participants were instructed to recall the last *n* letters after seeing a sequence of individually appearing letters which flashed on their screen. They were not told how many letters would be shown in total and had to recall letters in the order they appeared. For example, they were instructed to remember the last 2 letters, and the sequence “*X Y T R S*” appeared. The correct answer was “*R S*”. The number of letters to be recalled (*n)* ranged from three to seven, and sequences ranged from five to nine letters. The task contained five practice trials with feedback and 15 test trials without feedback. Accuracy on this test is a measure of working memory. Internal consistency estimates are excellent for accuracy on this test (0.85).

*Mini-IPIP* (Donnellan et al., 2006). This questionnaire presented participants with 20 statements and asked them to rate the degree to which they were an accurate description of them using a five-point rating scale. For example, participants rated “*Am the life of the party*” from being a *very inaccurate* (1) to *very accurate* (5) description of them. This scale measures the Big Five personality factors and has been shown to possess acceptable internal consistency for Agreeableness (0.70), Conscientiousness (0.69), Extraversion (0.77), Intellect (0.65), and Neuroticism (0.68).

#### Communication measures

We recorded the conversations between group members, while they completed the group tasks. Using these recordings, we computed the number of talking turns (frequency) to calculate equality of turn-taking. Equality of turn-taking, as defined by Woolley et al. (2010), refers to the standard deviation computed on the total number of talking turns for the members of a group. In the current study, zero indicated equality as both group members had the same number of talking turns, and higher values indicated greater inequality. To aid interpretation, we labelled this variable *inequality of turn-taking.* This variable was computed separately for each test in the CI battery and overall.

### Procedure

All participants were randomly assigned to dyads when they arrived at the university computer lab. Up to four participants (two dyads) completed the two-hour study at a testing session. Group members were seated at computers next to each other. Computer screens were arranged so that group members could see each other but not each other’s screens. The order of tasks was counterbalanced to reduce the impact of practice or fatigue effects, and to prevent dyads completing the study at the same time from overhearing answers to the same task. After providing consent, all participants completed a demographic questionnaire, the cognitive tests, and Mini-IPIP. The same items were completed by individuals and dyads on the CRT and ADR tests. For these tests, participants answered a question alone then again with their teammate before moving onto the next question. When answering individually, the same item appeared on each group member’s screen. Participants indicated a response using their keyboard. Participants then typed how confident they were that their answer was correct. Participants were instructed to wait for their partner before proceeding to the group stage. When both group members were ready, they pressed the spacebar on their keyboards. The same item appeared on each member’s screen accompanied by instructions to “*Discuss your answer with your partner. Try to persuade them if necessary. Come to an agreement and give the same answer”.* After submitting the same answer, participants indicated how confident they were that their group answer was correct. They were instructed to answer this alone, without discussing their level of confidence with their partner. After submitting a confidence rating, the next question began. For RAPM and MDMT, participants completed matched versions of the tests as individuals and dyads. The two versions were completed as described above with an important difference: individual and group items were completed in separate blocks. The protocol was approved by the University of Sydney Human Research Ethics Committee (Project Number 2017/729).

## Results

### Descriptive statistics

*Accuracy and confidence.* All analyses, except internal consistency estimates, were based on dyads as the unit of analysis. Thus, “individual” results refer to the average of the two group members working alone. This approach aligns with previous research on dyadic decision-making (Bahrami et al., 2010; Bang et al., 2014; Blanchard et al., 2020; Koriat, 2015; Schuldt et al., 2017). The descriptive statistics and internal consistency estimates for the five measures of accuracy and confidence, for both individuals and dyads, are presented in Table 2. Omega total (McDonald, 1999) was used to measure internal consistency since we assumed unidimensionality but not tau-equivalence for each of the variables. The *t* tests examined differences between individuals and dyads across each variable for each test.

**Table 2:** Descriptive statistics, internal consistency estimates, and *t* tests comparing individuals and dyads on each test for measures of accuracy and confidence (*N* = 105)

	Individuals			Dyads			*t* value
	*ω*_*t*_	Mean	SD	*ω*_*t*_	Mean	SD	
Accuracy							
ADR	0.71	58.33	18.78	0.73	74.15	20.93	− 16.00^***^
CRT	0.75	47.65	23.41	0.78	70.25	29.43	− 16.43^***^
MDMT	0.82	55.03	19.54	0.80	86.43	16.84	− 17.15^***^
RAPM	0.83	68.88	15.68	0.65	79.51	12.84	− 10.96^***^
GT	0.43	71.01	11.63	0.42	75.00	13.48	− 4.30^***^
Confidence							
ADR	0.91	83.68	13.45	0.92	88.61	11.66	− 10.17^***^
CRT	0.80	74.03	15.04	0.81	83.69	13.83	− 11.52^***^
MDMT	0.97	63.83	18.38	0.97	87.14	13.36	− 15.52^***^
RAPM	0.92	71.34	13.55	0.92	76.77	13.32	− 6.33^***^
GT	0.93	72.72	9.76	0.87	76.77	10.74	− 9.34^***^

*ω*_*t*_ = Internal consistency measured using Omega total; ADR = Applying Decision Rules; CRT = Cognitive Reflection test; MDMT = Medical Decision-Making test; RAPM = Raven’s Advanced Progressive Matrices; GT = Geography Test

^***^p < 0.001

The means and standard deviations for individual accuracy and confidence were comparable with other studies that used the same measures with undergraduate populations (Blanchard et al., 2020; Jackson et al., 2016, 2017; Law et al., 2022). We also examined differences between individuals and dyads on accuracy and confidence. For each test, accuracy and confidence were higher for dyads than individuals. This is consistent with previous research, which found that groups tend to be more accurate (e.g. Bahrami et al., 2010; Henry, 1993; Hill, 1982; Tindale, 1989; Zarnoth & Sniezek, 1997) and confident (e.g. Blanchard et al., 2020; Koriat, 2015; Sniezek & Henry, 1989; Zarnoth & Sniezek, 1997) than individuals across a range of cognitive and decision-making tasks.

For accuracy, internal consistency ranged from acceptable (0.65) to excellent (0.83) for individuals and dyads across all tests except the geography test which demonstrated poor internal consistency for individual (0.43) and dyadic responses (0.42). For confidence, internal consistency was excellent ranging from 0.80 to 0.97.

When we included the geography test in the CFA model, the loading (0.21) and communality (0.04) values were poor suggesting that accuracy on the geography test shared minimal variance with the underlying CI factor and did not fit well into the model. Thus, the geography test was removed from all subsequent analyses. Refer to the supplementary materials for detailed results of this analysis.

*Individual Differences and Communication Measures.* Descriptive statistics and internal consistency measures are presented in the supplementary materials to maintain focus on the intelligence and confidence variables.

### Extracting collective intelligence and confidence factors

Using the same approach employed by Woolley et al. (2010), metrics of CI and collective confidence were estimated using CFA to extract latent factors from measures of accuracy and confidence recorded across the three CI tasks.

**Fig. 1:**
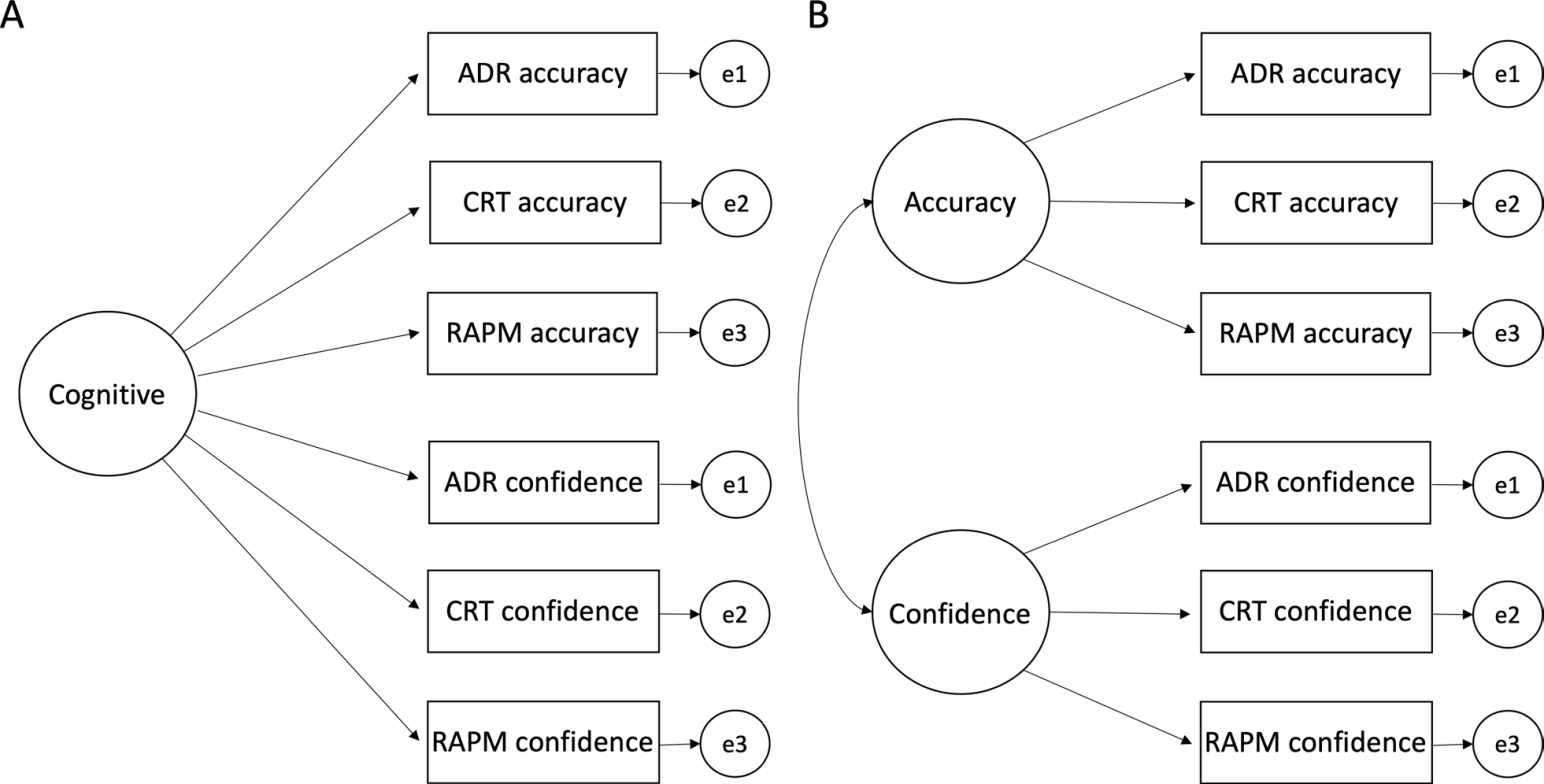
Hypothesised one first-order factor model (**A**) and two first-order factors model (**B**). Both models are without modification. solid lines represent positive loadings/correlations

We conducted CFA using the maximum likelihood method via the lavaan package (Rosseel, 2012) in R. We fitted and compared two first-order models that examined the factor structure of collective accuracy and confidence scores across the three tests for dyads. We tested hypothesised models with one first-order factor and two first-order factors (see Fig. 1). Each model was first tested without modification; thus, only hypothesised variables were allowed to define the respective factor, and correlations between latent constructs were freely estimated. Next, we tested modified versions of the same model to compare the fit indices. For these modified models, only the hypothesised variables were allowed to define the respective factor, but the error terms of accuracy and confidence derived from the same test were allowed to correlate because they were not independent and were derived from the same measure. See Fig. 1 for diagrams of the unmodified one- and two-factor models that were tested. Descriptions of the models, and a summary of fit indices, are presented in Table 3.

**Table 3:** Summary of fit indices evaluating different models of intelligence and confidence for dyads using maximum likelihood CFA (*N* = 105)

Model	Fit statistics									
	*R*^2^	*χ*^*2*^	*df*	*Χ*^*2*^*/df*	*χ*^*2*^ diff	GFI	TLI	CFI	RMSEA (90% CInt)	AIC
One-factor^a^	0.51	69.46	9	7.72	–	0.99	0.68	0.81	0.25 (0.20–0.31)	5014
Two-factor 1^b^	0.57	57.88	8	7.24	11.58^***^	0.99	0.70	0.84	0.24 (0.19–0.30)	5004
Two-factor 2^c^	0.58	16.72	7	2.39	41.17^***^	0.99	0.93	0.97	0.11 (0.04–0.19)	4966
Two-factor 3^d^	**0.57**	**6.23**	**6**	**1.04**	51.65^***^	**0.99**	**0.99**	**0.99**	**0.02 (0.00–0.13)**	**4957**

GFI = Goodness-of-fit index; CFI = Comparative Fit Index; TLI = Tucker–Lewis Index; RMSEA = Root-Mean-Square Error of Approximation; CInt = Confidence Interval; AIC = Akaike Information Criterion. The accepted model is in bold

^***^p < 0.001

^a^One-factor model consisted of one broad first-order cognitive factor defined by all the measures employed in the study without any modifications to the model

^b^Two-factor model consisted of an intelligence factor (defined by all accuracy measures) and a confidence factor (defined by all confidence measures) without any modifications to the model

^c^Two-factor model (intelligence and confidence factors) where error terms of the corresponding accuracy and confidence scores from RAPM were correlated

^d^Two-factor model (intelligence and confidence factors) where error terms of the corresponding accuracy and confidence scores from RAPM and CRT were correlated

A modified two-factor model had the best fit for collective accuracy and confidence (model 3^d^). In this model, the error terms of accuracy and confidence were correlated within the same test for RAPM and CRT. The Pearson correlation between accuracy and confidence was 0.46 (*p* < 0.001) for ADR, 0.66 (*p* < 0.001) for CRT, and 0.68 (*p* < 0.001) for RAPM. When the error terms were correlated across all 3 tests, the model was overfitted with Tucker–Lewis index exceeding 1, so we decided to omit the variables from the test with the smallest correlation (i.e. ADR). The fit indices for this modified two-factor model were excellent: *R*^2^ = 0.57; *χ*^2^/*df* = 1.04; goodness-of-fit index (GFI) = 0.99; Tucker–Lewis index (TLI) = 0.99; comparative fit index (CFI) = 0.99; and root-mean-square error of approximation (RMSEA) = 0.02 (CI 0.00–0.13). The results of this CFA model are displayed in Table 4. More detailed results of this model and the other two-factor models tested are presented in the supplementary materials.

**Table 4:** Summary of standardised regression weights, communalities, and correlations from a CFA using dyadic variables (*N* = 105)

Measures	Intelligence	Confidence	*h*^*2*^
ADR accuracy	0.60		0.35
CRT accuracy	0.86		0.73
RAPM accuracy	0.65		0.42
ADR confidence		0.80	0.63
CRT confidence		0.93	0.86
RAPM confidence		0.68	0.47
*Factor intercorrelations*			
Intelligence	1	0.85^***^	
Confidence		1	

All loadings and the factor intercorrelation were significant with *p* < 0.001

The extracted factors were interpreted as follows:

Factor 1, *CI*: As hypothesised, this factor was defined by the loadings of all accuracy scores on the three tasks: ADR, CRT, and RAPM. These measures are all known to capture cognitive ability, thereby defining a CI trait.

Factor 2, *collective confidence*: As hypothesised, this factor was defined by loadings of all confidence scores on the three tasks. This factor captured a collective metacognitive confidence trait.

There was a strong, positive correlation between individual intelligence and CI (*r* = 0.65, *p* < 0.001), and individual confidence and collective confidence (*r* = 0.70, *p* < 0.001). There was also a strong correlation between the CI and collective confidence factors (*r* = 0.85, *p* < 0.001) which might suggest a one-factor model; however, the CFA model with one-factor had poor fit indices (see Table 3).

### The predictors of collective intelligence and confidence

To examine our first three hypotheses, we conducted a hierarchical regression analysis. The independent variables included in the model were individual intelligence, individual confidence, social sensitivity, inequality of turn-taking, the number of female members, working memory accuracy, and Big Five personality.^1^ The number of female members was treated as a categorical variable with three levels: all male dyads (baseline), mixed gender dyads, and all female dyads. Consistent with the approach employed by Woolley et al. (2010), individual metrics of intelligence and confidence were represented by mean accuracy and confidence scores measured with RAPM. Woolley et al.’s key variables were included in block 1, the control variables in block 2, and individual intelligence and confidence were added in the final block. To examine, hypothesis four, the same hierarchical regression model was fit with collective confidence as the outcome variable. See Table 5 for a summary of the results.

**Table 5:** Results of hierarchical regression analyses using individual variables to predict collective intelligence and confidence

	Collective Intelligence			Collective Confidence		
	Block			Block		
	1	2	3	1	2	3
Predictor	β	β	β	β	β	β
Mixed gender dyads	− 0.15	− 0.18	− 0.14	− 0.29	− 0.27	− 0.28
Female dyads	− 0.85^**^	− 0.82^**^	− 0.54^*^	− 1.08^***^	− 1.05^***^	− 0.64^**^
Social sensitivity	0.39^***^	0.34^***^	0.22^**^	0.28^**^	0.20^*^	0.13
Inequality of turn-taking	0.09	0.08	0.08	0.14	0.11	0.12
WM accuracy	–	0.23^*^	0.10	–	0.33^***^	0.22^**^
Agreeableness	–	− 0.02	0.03	–	0.11	0.13
Conscientiousness	–	− 0.01	− 0.04	–	0.06	− 0.02
Extraversion	–	− 0.20^*^	− 0.09	–	− 0.01	0.07
Intellect	–	0.08	− 0.09	–	− 0.03	− 0.22^**^
Neuroticism	–	0.01	− 0.04	–	0.01	− 0.02
Intelligence	–	-	0.33^**^	–	-	− 0.02
Confidence	–	-	0.32^***^	–	-	0.63^***^
*R*	0.48	0.59	0.77	0.50	0.59	0.79
*R*^2^	0.23	0.35	0.60	0.25	0.35	0.63
Δ*R*^2^	0.23^***^	0.12^*^	0.25^***^	0.25^***^	0.10^*^	0.28^***^

WM = Working memory. *β* = Standardised regression coefficient

^***^p < 0.001, ^**^ *p* < 0.01, ^*^ *p* < 0.05

*Collective intelligence.* In block 1, social sensitivity (*β* = 0.39, *p* < 0.001) and all female dyads (*β* = − 0.85, *p* < 0.01) were significant predictors, accounting for 23% of the variance in CI. In block 2, working memory accuracy and the Big Five personality traits together accounted for an additional 12% of the variance in CI (Δ*R*^2^ = 0.12, *p* = 0.01). In block 3, individual intelligence (*β* = 0.33, *p* < 0.01) and individual confidence (*β* = 0.32, *p* < 0.001) accounted for an additional 25% of variance in CI (Δ*R*^2^ = 0.25, *p* < 0.001). In support of hypotheses 1 and 3, individual intelligence and confidence were significant positive predictors of CI, and both were stronger predictors than social sensitivity, inequality of turn-taking, and the gender composition of dyads. Furthermore, in support of hypothesis 2a, female dyads had significantly lower CI than male dyads (*β* = − 0.54, *p* = 0.01); however, there was no difference between male dyads and mixed gender dyads (*β* = − 0.14, *p* = 0.47). Hypothesis 2b was partially supported: equality of turn-taking did not predict CI (*β* = 0.08, *p* = 0.24), but social sensitivity was a significant positive predictor (*β* = 0.22, *p* < 0.01).

*Collective confidence.* In block 1, social sensitivity (*β* = 0.28, *p* < 0.01) and female dyads (*β* = − 1.08, *p* < 0.001) were significant predictors, accounting for 25% of the variance in CI. In block 2, working memory accuracy and the Big Five personality traits together accounted for an additional 10% of variance in CI (Δ*R*^2^ = 0.10, *p* = 0.04). In block 3, individual intelligence (*β* = − 0.02, *p* = 0.87) and individual confidence (*β* = 0.63, *p* < 0.001) accounted for an additional 28% of the variance in CI (Δ*R*^2^ = 0.28, *p* < 0.001). The significant predictors of collective confidence were individual confidence, the number of females, working memory accuracy, and intellect. In contrast, the significant predictors of CI were individual intelligence, individual confidence, social sensitivity, and the number of females. In support of hypothesis 4, the pattern of significant relationships differed for CI and collective confidence.

### Mediation analysis for the proportion of females, social sensitivity, and collective intelligence

A mediation analysis was conducted to examine whether social sensitivity mediated the relationship between proportion of females and CI. We conducted this analysis using the same data and predictors as block 3 of the hierarchical regression models. We used nonparametric bootstrapping with 1,000 simulations to generate confidence intervals. The results are presented in Table 6.

**Table 6:** The results of a mediation analysis for social sensitivity mediating the relationship between proportion of females and CI

Effect	95% CI		
	Estimate	Lower	Upper
Indirect (ACME)	0.21^**^	0.05	0.42
Direct (ADE)	− 0.54^*^	− 0.89	− 0.15
Total	− 0.33	− 0.69	0.06
Proportion mediated	− 0.62	− 6.07	3.23

^**^p < 0.01, ^*^p < 0.05

We found a significant positive indirect effect (ACME = 0.21, *p* < 0.01), indicating that social sensitivity significantly mediated the effect of the proportion of females on CI. This suggests that dyads with a higher proportion of females tended to have higher levels of social sensitivity which was associated with higher CI. We also observed a significant negative direct effect (ADE = − 0.54, *p* = 0.01), suggesting that dyads with a higher proportion of females had lower CI, independent of social sensitivity. This was supported by small negative correlations between the proportion of females and dyadic accuracy on each of the tests used to measure CI: Applying Decision Rules (*r* = − 0.11, *p* = 0.25), Cognitive Reflection Test (*r* = − 0.25, *p* < 0.01), and RAPM (*r* = − 0.10, *p* = 0.32).

The total effect of the proportion of females on CI approached but did not reach significance (Total Effect = − 0.33, *p* = 0.11), likely because the indirect and direct effects were in opposite directions and partially cancelled each other out. The proportion of the effect mediated was − 0.62 (*p* = 0.11) which also approached but did not reach significance. The wide confidence interval suggests instability in this estimate.

These results indicate that social sensitivity partially accounted for the relationship between the proportion of females and CI. However, the negative direct effect between the proportion of females and CI suggests a complex relationship that depends on factors beyond social sensitivity.

### Latent profile analysis

We then used individual and collective measures of intelligence, confidence, and bias to identify unique psychological profiles of dyads that clustered together on these measures. LPA was used to classify the observations under study into distinct profiles given their homogenous characteristics across a set of estimated values for the predictor variables.

#### Selecting an LPA solution

**Fig. 2:**
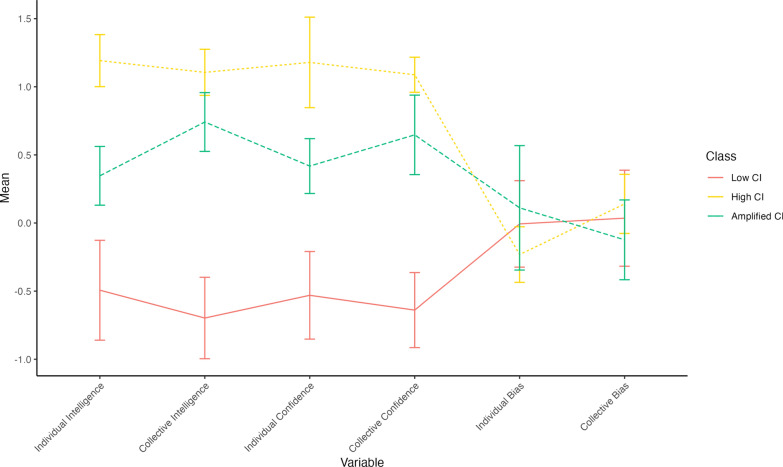
Latent profile dyads for the 3-class solutions. Error bars represent the standard error of the mean for each profile on each variable

LPA was performed for solutions with 2–6 classes on six predictor variables. These variables were individual intelligence and confidence, the extracted factors for CI and collective confidence, and individual and collective bias scores. Goodness-of-fit statistics were used to identify the number of latent profiles (Clark & Muthén, 2009; Henson et al., 2007; Marsh et al., 2009). Assessment of the indices and examination of the profiles within each model suggested a 3-Class solution was the best fitting model (see Fig. 2). Refer to the supplementary materials for a detailed summary of this assessment process.

#### Interpretation of the 3-class solution

The percentage of participants in each of the three classes was as follows: 54.29% in Class 1 (*n* = 57), 14.29% in Class 2 (*n* = 15), and 31.43% in Class 3 (*n* = 33). The three distinct profiles differed significantly on individual intelligence, CI, individual confidence, and collective confidence. The profiles were interpreted as follows: (1) *low CI*: Those who were low on intelligence and confidence and well calibrated at both the individual and collective levels; (2) *high CI*: Those who were high on intelligence and confidence and well calibrated at the individual and collective levels; and (3) *amplified CI*: Those who were moderate on individual intelligence and significantly higher on CI, moderate on individual and collective confidence, and well calibrated at both levels. Using the Bonferroni correction, amplified CI (profile 3) had significantly higher CI than Individual intelligence (*t*_32_ = 45.08, *p* < 0.001). Furthermore, high CI had significantly higher collective bias than individual bias (*t*_14_ = 3.35, *p* < 0.01). There were no other differences between individual and collective scores within the profiles.

#### Differences between the three profiles

First, a MANOVA was conducted to test whether the three profiles differed across social sensitivity, inequality of turn-taking, the number of females, working memory accuracy, and Big Five personality traits. A MANOVA indicated that the three profiles significantly differed across the theoretically relevant outcome variables (*F*_18,176_ = 2.52, Wilk’s *Λ* = 0.63, *p* < 0.01, *η*_*p*_^2^ = 0.21).

**Fig. 3:**
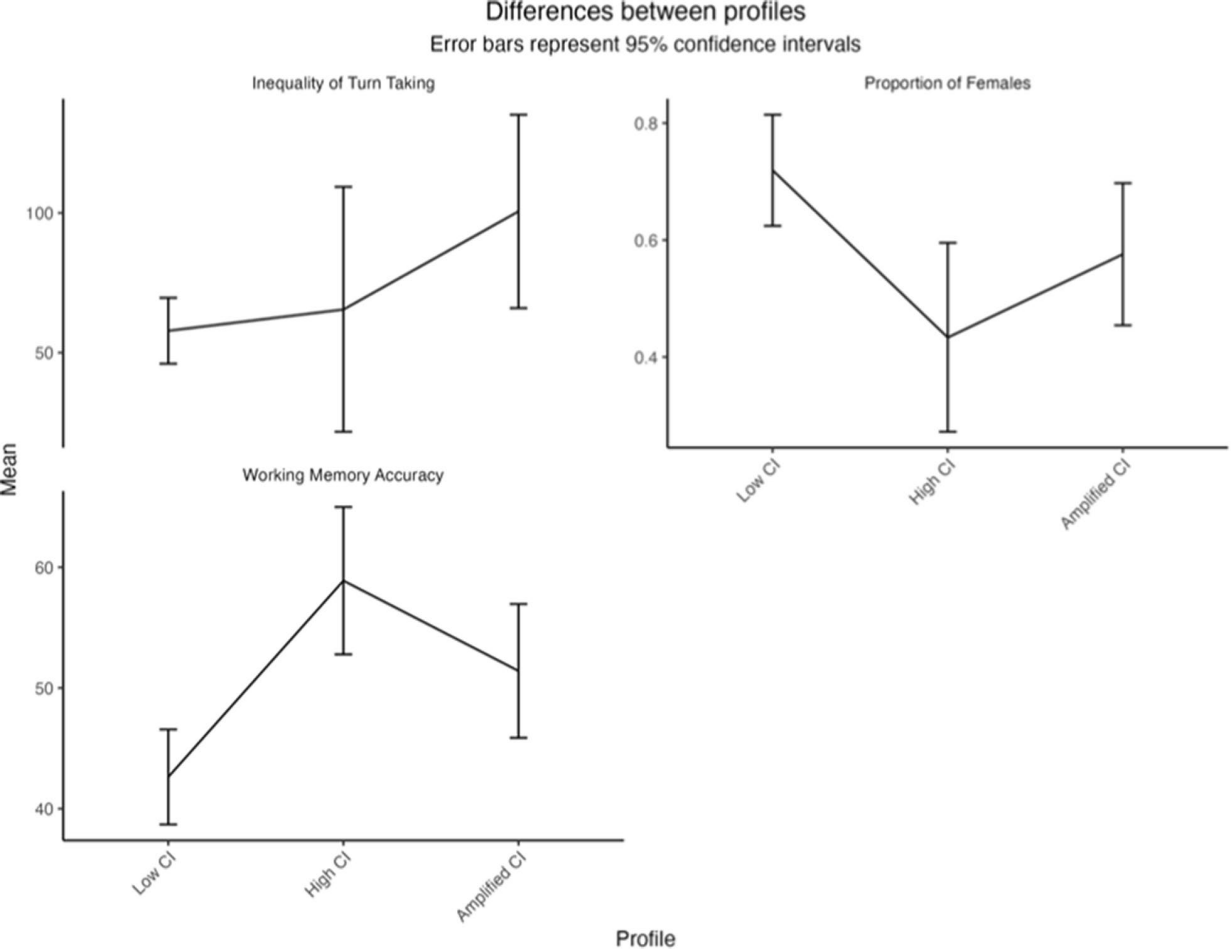
The demographic, psychological, and communication variables that significantly differed between the three profiles

Next, univariate ANOVAs were conducted to identify differences between the profiles on the relevant outcome variables. See Table 7 and Fig. 3 for a summary of these analyses. The series of ANOVAs showed that the three profiles significantly differed on inequality of turn-taking (*F*_2,96_ = 3.11, *p* = 0.04, η_p_^2^ = 0.06), the number of females (*F*_2,96_ = 3.91, *p* = 0.02, η_p_^2^ = 0.08), and working memory accuracy (*F*_2,96_ = 8.43, *p* < 0.001, *η*_*p*_^2^ = 0.15). Although, the differences, including the effect sizes, were moderate to small, with partial eta squared values ranging between 0.06 and 0.15.

**Table 7:** Results of univariate ANOVA TESTS WITH CONTRASTS FOR THE DIFFERENCES BETWEEN PROFILES ON THE DEMOGRAPHIC AND PSYCHOLOGICAL VARIABLES

Measure	Mean Low CI (SD)	Mean High CI (SD)	Mean amplified CI (sd)	*F*_2,99_	η_p_^2^	c1–2	c1–3	c2–3
Woolley et al. (2010)* variables*								
Social sensitivity	55.65 (10.26)	58.43 (8.73)	59.57 (9.20)	1.65	0.03	0.59	0.20	0.93
Inequality of turn-taking	57.59 (45.77)	58.43 (8.73)	99.27 (103.06)	3.11^*^	**0.0**4	0.58	**0.04**	0.32
Number of females	0.71 (0.37)	0.43 (0.32)	0.61 (0.34)	3.91^*^	**0.08**	**0.02**	0.40	0.28
*Other variables*								
Working memory accuracy	42.50 (15.27)	58.89 (12.06)	51.67 (16.04)	8.43^***^	**0.15**	** < 0.001**	**0.03**	0.30
Agreeableness	4.02 (0.44)	3.91 (0.49)	3.92 (0.55)	0.58	0.01	0.70	0.64	0.99
Conscientious	3.24 (0.68)	3.13 (0.51)	3.17 (0.62)	0.23	0.01	0.82	0.90	0.97
Extraversion	3.03 (0.60)	2.69 (0.63)	2.92 (0.62)	1.92	0.04	0.14	0.68	0.49
Intellect/Openness	3.60 (0.52)	3.78 (0.60)	3.75 (0.60)	1.01	0.02	0.51	0.49	0.98
Neuroticism	2.96 (0.51)	3.18 (0.47)	2.91 (0.49)	1.59	0.03	0.26	0.92	0.21

CI = collective intelligence. *η*_*p*_^2^ = partial eta squared. *c* = contrast. Contrasts were tested using the Tukey–Kramer post hoc test. Significant contrasts and relevant eta squared values are in bold

*p < 0.05; **p < 0.01; ***p < 0.001

Lastly, because there was an unequal number of members in each profile, Tukey–Kramer post hoc tests were conducted on significant outcome variables to examine differences between the three profiles while controlling for multiple comparisons. Post hoc tests revealed that the high CI profile had a significantly lower number of females (*p* = 0.02) and higher working memory accuracy (*p* < 0.001) compared with the low CI profile. Furthermore, the amplified CI profile had significantly higher inequality of turn-taking (*p* = 0.04) and working memory accuracy (*p* = 0.03) compared with the low CI profile. The high and amplified CI profiles did not differ on any of the variables.

## Discussion

The purpose of this study was to enhance the operationalisation of CI by employing a novel methodology for both measurement and analyses. Departing from previous research that relied on a theoretical taxonomy, the McGrath Task Circumplex, we used the empirically validated CHC model to guide the selection of well-structured tasks. This approach allowed us to assess the cognitive abilities relevant to CI. Our measure of CI captured three broad abilities: Fluid Reasoning, Crystallised Intelligence, and Quantitative Knowledge. We then examined the relationships between dyadic CI, individual intelligence, individual confidence, and other key characteristics. Additionally, we conducted a mediation analysis to investigate whether social sensitivity meditated the relationship between the proportion of females and CI. Next, we utilised LPA to identify unique psychological profiles of dyads derived from measures of intelligence, confidence, and bias. Finally, we assessed differences between these psychological profiles across several characteristics relevant to dyadic CI.

### The predictors of collective intelligence for well-structured tasks

Our results demonstrate that individual intelligence is a strong predictor of CI in dyads for well-structured tasks. Furthermore, individual confidence also emerged as a strong predictor of CI, contributing uniquely beyond individual intelligence. These findings contrast with those of Woolley and colleagues using larger groups, who reported a weak correlation between individual intelligence and CI and emphasised the importance of social and compositional factors. By addressing previous methodological limitations, we have shown that smarter and more confident individuals form dyads with higher CI.

Currently, there is no consensus on how CI should be conceptualised or measured. Some researchers, such as Woolley et al. (2010), adopt a broad definition that includes a variety of task types and emphasises emergent group processes such as coordination and communication. Graf-Drasch et al. (2022) proposed that different conceptualisations of CI may be required for well- and ill-structured tasks, guided by the findings that different cognitive and coordination processes are utilised for each task type. For well-structured tasks, general cognitive and coordination processes relate to performance, whereas ill-structured tasks may rely on task-specific processes (Newell & Simon, 1972; Schraw et al., 1995; Simon, 1973). Our approach, along with that of Rowe et al. (2024), builds upon this. We focus more narrowly than Woolley and colleagues on identifying the cognitive underpinnings of dyadic performance using well-structured tasks informed by models of individual intelligence. This approach allows for the systematic isolation and testing of how specific cognitive abilities contribute to group outcomes. It is not intended to provide a comprehensive account of CI but rather serve as a theoretically driven starting point for building a broader model of CI for well-structured tasks. Although our tasks required communication and coordination, thus capturing some key aspects of group interactions, we did not score these processes as outcomes, as in Woolley et al.’s work. We view these approaches as complementary rather than contradictory. Acknowledging these differing perspectives is essential for developing integrative models that specify how cognitive and interactional processes contribute to collective performance across different task contexts.

Our study focused on well-structured tasks, and we found that a single CI factor is appropriate for these tasks. In contrast, ill-structured tasks, which feature ambiguous pathways and multiple possible solutions, may require a multi-factor conceptualisation of CI as suggested by Graf-Drasch et al. (2022). We did not assess CI for these tasks so we can only speculate that the presence of ill-structured tasks reduces a group’s reliance on cognitive abilities and increases the importance of coordination processes for performance. This is supported by Woolley et al.’s (2010) results which are largely based on ill-structured tasks, where CI was predicted by factors related to coordination and social interaction (i.e. social sensitivity, equality of turn-taking, and the number of females). Therefore, Woolley and colleagues’ concept of CI may more accurately reflect teamwork processes and dynamics than the cognitive abilities driving group performance (Graf et al., 2019; Hackman & Morris, 1975; LePine et al., 2008; Rowe et al., 2021). Ill-structured tasks are representative of many real-world scenarios encountered by groups; thus, understanding the task dependency of cognitive and coordination processes in CI is a critical question for future research.

There are several reasons why our findings diverge from those of Woolley et al. (2010). First, we addressed methodological limitations by using the empirically validated CHC model to guide task selection, rather than relying on theoretical taxonomies, such as the McGrath Group Task Circumplex, which may not adequately capture the cognitive abilities relevant to CI (Bell, 2007; Rowe et al., 2021). Second, by focusing exclusively on well-structured tasks, we avoided the confounding effects of mixing task types that require different cognitive and coordination processes. This allowed us to more precisely assess the role of individual intelligence in CI for well-structured tasks. Future research should examine CI in ill-structured tasks and a mix of well- and ill-structured tasks together. All are valid and important approaches, as many real-world projects are likely to contain subtasks that may blend both task types. Third, the difference in group size may have influenced the results. Our study, used dyads, whereas prior research typically involved groups with 2 to 5 members (e.g. Barlow & Dennis, 2016; Bates & Gupta, 2017; Engel et al., 2014; Rowe et al., 2024; Woolley et al., 2010). The group dynamics that exist in dyads may emphasise cognitive abilities more than in larger groups, where coordination and interaction processes become more complex. Future research should investigate whether our findings apply to larger groups.

In addition to individual intelligence and confidence, we found that several other characteristics predicted CI. Consistent with Woolley et al. (2010), higher social sensitivity was associated with higher CI, and the proportion of females had a positive indirect relationship with CI through social sensitivity. We used a different measure of social sensitivity because the original test (Reading the Mind in the Eyes) has been shown to have several critical limitations (e.g. Black, 2017; Kittel et al., 2022; Olderbak et al., 2015). Thus, these findings support their claims that the proportion of females and social sensitivity are important for CI beyond cognitive abilities. However, we also found a strong direct negative relationship between the proportion of females and CI, indicating that, independent of social sensitivity, dyads composed of two females had lower CI scores than male only dyads but not mixed gender dyads. This divergence from Woolley et al. indicates that factors other than social sensitivity may be influencing the relationship between the proportion of females and CI in dyads performing well-structured tasks.

One possible explanation for this discrepancy lies in the cognitive demands of the tasks used to assess CI. Our tasks primarily assessed cognitive abilities, such as Fluid Reasoning and Quantitative Knowledge, that tend to confer a small performance advantage to males (Halpern et al., 2007; Irwing & Lynn, 2005; Otero et al., 2024). Supporting this, we found small to negligible negative correlations between the proportion of females and accuracy on each of the tests used to measure dyadic CI. These findings suggest that the cognitive abilities required by our well-structured tasks may have contributed to lower CI scores in dyads with a higher proportion of females. Another possibility is that the simpler social dynamics found in dyads compared to larger groups may reduce the influence of social sensitivity. In dyads, communication and coordination are more straightforward than in larger groups, and as a result these factors may play a diminished role in our study compared to Woolley et al. and others that used larger groups. This could also account for our failure to replicate the positive relationship between equality of turn-taking and CI. Furthermore, higher working memory accuracy was associated with higher CI, indicating that the capacity to hold and manipulate information in one’s mind is important for dyadic CI on well-structured tasks.

As expected, the pattern of variables that predicted CI was different from those that predicted collective confidence. This finding supports Koriat’s (2024) claim that measures of confidence provide unique information beyond accuracy. Confidence may reflect the consistency with which individuals or dyads would respond to the same task again in the future, not merely the correctness of their responses. This finding highlights the importance of considering confidence as a separate construct influencing dyadic performance.

### The psychological profiles of dyads

The application of LPA in our study allowed us to identify three distinct psychological profiles of dyads: (1) low CI profile (approximately 54% of dyads); (2) high CI (approximately 14%); and (3) amplified CI (approximately 32%). This person-centred approach is novel in the study of CI and provides insights into how combinations of individual intelligence and confidence contribute to dyadic performance. The high CI profile was comprised of dyads with two high individual intelligence and confidence members, leading to high CI. Furthermore, this profile had higher working memory accuracy and fewer females than the low CI profile. The amplified CI profile consisted of dyads where CI exceeded individual intelligence, suggesting that collaboration led to performance improvements. Interestingly, amplified CI dyads had higher working memory accuracy and greater inequality of turn-taking compared to low CI dyads, indicating that enhanced dyadic collaboration may involve strategic dominance by the more knowledgeable member. This contrasts with Woolley et al. (2010), who found that equality of turn-taking predicted higher CI in larger groups. Our findings suggest that for dyads completing well-structured tasks, allowing the more competent member to lead may enhance collective performance. These results highlight the importance of considering individual intelligence, confidence, and possibly working memory and interaction patterns when forming dyads. Working memory accuracy and inequality of turn-taking did not differ between high and amplified profiles, so they may not offer comparable precision as intelligence and confidence.

### Implications, limitations, and future directions

Our findings have important implications for the measurement of CI, professional practice, and future research. Individual intelligence and confidence emerged as key predictors of CI on well-structured tasks. This suggests that forming dyads with smart and confident individuals may enhance dyadic performance without the need for a specific measure of CI. However, our findings are limited to dyads and may not generalise to larger groups.

As group size increases, Woolley et al.’s (2010) key predictors of CI (social sensitivity, equality of turn-taking, and proportion of females) may play a larger role as group dynamics like coordination and interaction processes become more complex. Future research should investigate CI and Woolley’s key predictors using well-structured tasks and larger groups.

A specific measure of CI, as developed by Woolley et al. (2010), may be more useful for ill-structured tasks which tend to rely on different cognitive and coordination processes than well-structured tasks. For these tasks, group interaction processes may be more important than cognitive abilities. Since many real-world tasks are ill-structured, future research should examine CI for these tasks separately. Preliminary research indicates that CI in ill-structured tasks may be represented by multiple factors that capture characteristics of coordination and interaction rather than cognitive abilities (e.g. Graf‐Drasch et al., 2022; LePine et al., 2008). This raises an interesting question for future research: if coordination processes are more important for CI on ill-structured tasks, can they be trained to improve CI?

In professional settings, dyadic performance on well-structured tasks can be enhanced by selecting individuals with higher intelligence, confidence, and social sensitivity. Our findings suggest that organisations should consider incorporating cognitive and metacognitive assessments into their hiring and team formation processes to identify individuals who are likely to contribute effectively in dyadic collaborations.

Furthermore, the results of the LPA indicate that strategic dominance by the more knowledgeable or competent member may enhance performance in dyads with moderate individual intelligence. This suggests that dyads could be trained to identify and leverage the strengths of the more competent member. Training programmes could focus on developing skills for recognising expertise and allowing the more competent member to lead discussions and decision choices in a strategic way. It remains unclear whether members should assess competence at the task level (analogous to individual tests in our study) or at the project level (analogous to our battery of tasks). This presents an open question for future research which could improve dyad training and formation strategies.

Our findings should be interpreted alongside several additional limitations. While using three tests, as we did, is a recommended minimum to measure a latent trait like intelligence, a larger number of tests could provide more robust measurement. We had a fourth measure, but the geography test demonstrated poor internal consistency and did not load on the CI factor when we fit a CFA model; thus, it was removed from our analyses. The remaining tasks captured three of 16 broad cognitive abilities proposed under the CHC model of intelligence (e.g. McGrew, 2009). Future research should investigate CI using a larger battery of well-structured tasks that cover more of these broad abilities.

Our analyses were conducted at the dyad level, requiring us to transform individual-level variables, such as individual intelligence and social sensitivity, into a single score representing the two original scores of the dyad members. There are several methods for representing individual-level constructs at the dyad level, each with potential information loss that may be important for group dynamics: 1) the average score, which includes information from both members but reduces within-dyad variance; (2) the maximum score, which emphasises one member’s contribution but omits the other’s; and (3) a difference score or similarity metric, which preserves within-dyad variance but discards the magnitude of the original scores. For our analyses, we used the average to represent all individual-level variables at the dyad level. While this approach, and the alternatives, produces trade-offs, our method is widely accepted in group decision-making research (e.g. Blanchard et al., 2020; Koriat, 2015; Rowe et al., 2021; Schuldt et al., 2017). This issue was outside of the scope of this study. Future studies should systematically compare and contrast these and alternative methods to identify the best method of representing individual constructs at dyadic level.

An additional limitation is that we measured CI in naïve dyads who worked together for the first-time during our study. Prolonged collaboration may increase the impact of certain individual characteristics, such as personality traits, on dyadic dynamics. Moreover, the accumulated knowledge of individual members’ abilities could moderate the relationships between key predictors and CI. However, consistent with Woolley et al. (2010), we measured individual intelligence using a single test that captured Fluid Reasoning. This was adequate for our study, but future research should use a more comprehensive measure of individual intelligence. This may have the added benefit of clarifying the relationship between social sensitivity and CI.

### Conclusion

Our study introduces a novel approach to examining dyadic CI on well-structured tasks by utilising the CHC model for task selection and incorporating metacognitive confidence. We found that individual intelligence and confidence are stronger predictors of dyadic CI than social sensitivity and interaction processes like equality of turn-taking, which contrasts with previous findings by Woolley et al. (2010). Notably, only one of Woolley et al.’s findings for the three key predictors (social sensitivity) was replicated in our study. These results challenge the generalisability of prior CI measures to dyads and highlight the importance of cognitive abilities and confidence in enhancing collective performance on well-structured tasks. Our findings have important implications for the selection and training of dyads, suggesting that focusing on individual intelligence, confidence, and social sensitivity may be effective at improving dyadic performance.

## Supplementary Information

Supplementary material 1.

## Data Availability

The raw data supporting the conclusions of this article will be made available upon request, without undue reservation. The experiment described in this manuscript was not preregistered.

## Abbreviations

AIC: Akaike information criterion
ADR: Applying decision rules
CI: Collective intelligence
CRT: Cognitive Reflection Test
CFA: Confirmatory factor analysis
CHC: Cattell–Horn–Carroll
CFI: Comparative Fit Index
Cint: Confidence interval
GFI: Goodness-of-Fit Index
LPA: Latent profile analysis
MDMT: Medical decision-making
RAPM: Raven’s advanced progressive matrices
RMSEA: Root-mean-square error of approximation
SD: Standard deviation
TLI: Tucker–Lewis Index
WM: Working memory

## Footnotes

1 To retain power in the regression analyses, missing values were imputed using multiple imputation via the mice package in R (van Buuren & Groothuis-Oudshoorn, 2011). One participant had non-random missing data for 6 of the 12 predictors and thus was removed. The remaining missing data were random with less than < 1% for each predictor. For the focal variables, results were the same when analyses were conducted using the original and imputed data. See supplementary materials for the results using data without imputation.
