# Supplementary material for: A recipe for dyadic collective intelligence for well-structured tasks: mix equal parts cognitive ability and confidence plus a pinch of social sensitivity
Source: Cogn Res Princ Implic. 2025 Sep 24;10:63. doi: 10.1186/s41235-025-00655-0 (PMC12460217; doi:10.1186/s41235-025-00655-0)
Supplement: Supplementary file 1 — Supplementary material 1. [file 41235_2025_655_MOESM1_ESM.docx]

**Appendix A**

**Descriptive Statistics for Individual Differences and Communication Variables**

***Individual differences.*** The descriptive statistics and internal consistency estimates for the individual differences measures are reported in Table A1.

**Table A1**

*Descriptive Statistics* *and Internal Consistency Estimates for Individual Differences Measures (N=105)*

|  | N | ω_t_ | Mean | SD |
| --- | --- | --- | --- | --- |
| Social sensitivity | 103 | .71 | 57.28 | 9.66 |
| Working memory accuracy | 105 | .77 | 47.71 | 16.16 |
| Agreeableness | 104 | .71 | 3.94 | 0.50 |
| Conscientiousness | 104 | .73 | 3.20 | 0.64 |
| Extraversion | 104 | .74 | 2.94 | 0.62 |
| Intellect | 104 | .64 | 3.68 | 0.55 |
| Neuroticism | 104 | .62 | 2.95 | 0.51 |

*Note*: ω_t_ = Internal consistency measured using Omega total; SD = Standard Deviation.

The means and standard deviations for social sensitivity, working memory accuracy, and personality were comparable with other studies that have used the same measures on an undergraduate population (Jackson et al., 2017; Law et al., 2018; Law et al., 2022). Internal consistency estimates ranged from acceptable (.66) to excellent (.89) for all measures.

***Communication measures.*** Table A2 describes the descriptive statistics for the communication metrics captured during each test and overall. These variables were based on frequency counts thus, no estimate of internal consistency could be made.

**Table A2**

*Descriptive statistics* *for communication measures for each test and overall (N=101)*

| Test | Items | Number of talking turns | | | | Duration of discussion | | | |
| --- | --- | --- | --- | --- | --- | --- | --- | --- | --- |
|  |  | Total | | Equality | | Total (seconds) | | Equality | |
|  |  | Mean | SD | Mean | SD | Mean | SD | Mean | SD |
| ADR | 10 | 138.88 | 58.00 | 23.81 | 22.32 | 370.89 | 231.29 | 79.03 | 72.29 |
| CRT | 7 | 126.11 | 44.45 | 18.71 | 19.50 | 351.15 | 169.46 | 72.56 | 61.24 |
| RAPM | 18 | 348.81 | 153.21 | 41.56 | 40.01 | 1076.23 | 738.57 | 171.73 | 159.37 |
| Overall |  | 613.80 | 204.93 | 71.23 | 73.80 | 1798.26 | 980.05 | 279.58 | 270.03 |

*Note.* ADR = Applying Decision Rules, CRT = Cognitive Reflection Test; RAPM = Raven’s Advanced Progressive Matrices.

The CRT had the fewest number of speaking turns, the shortest duration of discussion, the greatest equality of turn taking, and the greatest equality of duration, on average. Raven’s Advanced Progressive Matrices, on the other hand, had the highest scores on each variable. This was not surprising given that it had the largest number and, arguably, the most complex items.

**Appendix B**

**Additional Results for CFA Without Geography Test**

Additional analyses were conducted to determine the best fitting CFA model. Table B1 reports the modification indices for the relationship between accuracy and confidence within each test for each model tested. Scores below ten are optimal. Tables B2, B3, and B4 report the standardised residual covariances for the models tested. Values greater than ±2.00 suggest poor model fit. The modification indices and standardised residual covariances indicate that the best fitting model was the modified two factor model with error terms correlated for RAPM and CRT.

**Table B1**
*Modification Indices for the Relationship Between Accuracy and Confidence for each test for the Unmodified Two Factor Model*

| Model | U2 | M2R | M2RC |
| --- | --- | --- | --- |
| RAPM | 35.76 | - | - |
| CRT | 15.73 | 10.02 | - |
| ADR | 6.56 | 8.68 | 3.59 |

*Note.* U2 = Unmodified two factor model. M2R = Modified two factor model with the error terms for RAPM correlated. M2RC = Modified two factor model with the error terms for RAPM and CRT correlated. RAPM = Raven’s Advanced Progressive Matrices. CRT = Cognitive Reflection Test. ADR = Applying Decision Rules.

**Table B2**
*Standardised Residual Covariances for the Unmodified Two Factor Model*

|  | ADR Acc | CRT Acc | RAPM Acc | ADR Conf | CRT Conf | RAPM Conf |
| --- | --- | --- | --- | --- | --- | --- |
| ADR Acc | 0.07 |  |  |  |  |  |
| CRT Acc | 0.48 | 0.00 |  |  |  |  |
| RAPM Acc | -0.02 | -0.31 | 0.00 |  |  |  |
| ADR Conf | 1.16 | -2.36 | -1.26 | -0.15 |  |  |
| CRT Conf | -0.90 | 1.48 | -0.91 | 0.70 | 0.00 |  |
| RAPM Conf | -0.74 | -0.74 | 3.83 | 1.08 | -1.20 | 0.08 |

*Note.* RAPM = Raven’s Advanced Progressive Matrices. CRT = Cognitive Reflection Test. ADR = Applying Decision Rules. Acc = Accuracy. Conf = Confidence.

**Table B3**
*Standardised Residual Covariances for the Modified Two Factor Model with the Error Terms for RAPM Correlated*

|  | ADR Acc | CRT Acc | RAPM Acc | ADR Conf | CRT Conf | RAPM Conf |
| --- | --- | --- | --- | --- | --- | --- |
| ADR Acc | -0.03 |  |  |  |  |  |
| CRT Acc | -0.60 | 0.00 |  |  |  |  |
| RAPM Acc | 0.17 | -0.54 | -0.68 |  |  |  |
| ADR Conf | 1.51 | -2.04 | -0.51 | -0.13 |  |  |
| CRT Conf | -0.61 | 2.20 | -0.25 | -0.79 | 0.00 |  |
| RAPM Conf | -0.33 | -0.50 | -0.74 | 1.32 | -1.00 | -0.43 |

**Table B4**
*Standardised Residual Covariances for the Modified Two Factor Model with the Error Terms for RAPM and CRT Correlated*

|  | ADR Acc | CRT Acc | RAPM Acc | ADR Conf | CRT Conf | RAPM Conf |
| --- | --- | --- | --- | --- | --- | --- |
| ADR Acc | 0.05 |  |  |  |  |  |
| CRT Acc | -0.29 | 0.48 |  |  |  |  |
| RAPM Acc | -0.91 | 1.63 | 0.21 |  |  |  |
| ADR Conf | 0.63 | -0.93 | -0.74 | -0.14 |  |  |
| CRT Conf | -0.41 | 0.19 | 1.52 | -0.72 | -0.12 |  |
| RAPM Conf | -0.77 | 1.14 | 0.29 | -0.33 | 1.54 | 0.21 |

**Appendix C**

**CFA Results with Geography Test Included**

We fitted the same CFA models that are reported in the main paper with the addition of accuracy and confidence on the geography test (GT). The results are presented in Tables C1 and C2. A modified two-factor model had the best fit for collective accuracy and confidence (model 3^d^). In this model, the error terms of accuracy and confidence were correlated within the same test for RAPM and CRT. The fit indices for this modified two-factor model were excellent: *R*^2^ = .47; χ^2^/*df* = 1.22; Goodness of Fit Index (GFI) = 0.99; Tucker-Lewis Index (TLI) = 0.98; Comparative Fit Index (CFI) = 0.99; Root Mean Square Error of Approximation (RMSEA) = 0.05 (CI = .00-.10). The results of this CFA model are displayed in Table C1. Accuracy from ADR, CRT, and RAPM load well onto the CI factor, however, GT accuracy loads poorly (.21, *p* > .05) and has a low communality (.04). Together, these values indicate that GT accuracy shares little variance with the underlying CI factor, suggesting it does not integrate well into the model. Thus, it was removed from the model and excluded from the analyses.

**Table C1**

*Summary of Fit Indices Evaluating Different Models of Intelligence and Confidence for Dyads Using Maximum Likelihood CFA (N = 105)*

| **Model** | **Fit Statistics** | | | | | | | | | |
| --- | --- | --- | --- | --- | --- | --- | --- | --- | --- | --- |
|  | *R*^2^ | *χ^2^* | *df* | *Χ^2^ / df* | *χ^2^* diff | GFI | TLI | CFI | RMSEA (90% CInt) | AIC |
| One-factor^a^ | .42 | 94.65 | 20 | 4.73 | - | 0.99 | .71 | .79 | .19 (.15-.23) | 6619 |
| Two-factor 1^b^ | .47 | 78.94 | 19 | 4.15 | 15.71^***^ | 0.99 | .75 | .83 | .17 (.13-.21) | 6605 |
| Two-factor 2^c^ | .48 | 36.28 | 18 | 2.02 | 42.66^***^ | 0.99 | 0.92 | 0.95 | .10 (.05-.14) | 6564 |
| **Two-factor 3^d^** | **.47** | **20.66** | **17** | **1.22** | **15.62^***^** | **0.99** | **0.98** | **0.99** | **.05 (.00-.10)** | **6550** |

*Note.* GFI = Goodness-of-fit index; CFI = Comparative Fit Index; TLI = Tucker-Lewis Index; RMSEA = Root Mean Square Error of Approximation; CInt = Confidence Interval; AIC = Akaike Information Criterion. The accepted model is in bold.

^***^ *p* < .001

^a^One-factor model consisted of one broad first order Cognitive factor defined by all the measures employed in the study without any modifications to the model.

^b^Two-factor model consisted of an intelligence factor (defined by all accuracy measures) and a confidence factor (defined by all confidence measures) without any modifications to the model.

^c^Two-factor model (intelligence and confidence factors) where error terms of the corresponding accuracy and confidence scores from RAPM were correlated.

^d^Two-factor model (intelligence and confidence factors) where error terms of the corresponding accuracy and confidence scores from RAPM and CRT were correlated.

^***^ *p* < .001, ^**^ *p* < .01, ^*^ *p* < .05.

**Table C2**

*Summary of Standardised Regression Weights, Communalities, and Correlations from a CFA Using Dyadic Variables and Including (N = 105)*

| Measures | Intelligence | Confidence | *h^2^* |
| --- | --- | --- | --- |
| ADR accuracy | .68 |  | .46 |
| CRT accuracy | .73 |  | .54 |
| RAPM accuracy | .63 |  | .40 |
| GT accuracy | .21 |  | .04 |
| ADR confidence |  | .88 | .78 |
| CRT confidence |  | .84 | .71 |
| RAPM confidence |  | .69 | .48 |
| GT confidence |  | .59 | .35 |
| *Factor intercorrelations* |  |  |  |
| *Intelligence* | 1 | .78 |  |
| *Confidence* |  | 1 |  |

*Note.* All loadings and the factor intercorrelation were significant with *p* < .001 except for GT accuracy which did not reach significance.

**Table C3**
*Standardised Residual Covariances for the Modified Two Factor Model with the Error Terms for RAPM and CRT Correlated*

|  | ADR Acc | CRT Acc | RAPM Acc | GK Acc | ADR Conf | CRT Conf | RAPM Conf | GK Conf |
| --- | --- | --- | --- | --- | --- | --- | --- | --- |
| ADR Acc | 0.04 |  |  |  |  |  |  |  |
| CRT Acc | 0.21 | 0.62 |  |  |  |  |  |  |
| RAPM Acc | -0.54 | 1.79 | 0.32 |  |  |  |  |  |
| GT Acc | 0.3 | -0.8 | -0.63 | 0.00 |  |  |  |  |
| ADR Conf | 0.78 | -0.25 | -0.32 | 0.37 | -0.08 |  |  |  |
| CRT Conf | -0.12 | 0.44 | 1.73 | -0.58 | -0.55 | -0.02 |  |  |
| RAPM Conf | -0.59 | 1.38 | 0.45 | -0.71 | -0.28 | 1.38 | 0.31 |  |
| GT Conf | -2.11 | -1.86 | -1.34 | 0.99 | -0.05 | 0.12 | 0.54 | 0.01 |

**Appendix D**

Table D1 presents the results of the hierarchical regression analyses fit using data without imputation.

**Table D1**

*Results of Hierarchical Regression Analyses Using Individual Variables to Predict Collective Intelligence and Confidence*

|  | Collective Intelligence | | | Collective Confidence | | |
| --- | --- | --- | --- | --- | --- | --- |
|  | Block | | | Block | | |
|  | 1 | 2 | 3 | 1 | 2 | 3 |
| Predictor | β | β | β | β | β | β |
| Mixed gender dyads | -.15 | -.25 | -.17 | -.32 | -.33 | -.32 |
| Female dyads | -.84^***^ | -.89^***^ | -.57^*^ | -1.12^***^ | -1.10^***^ | -.69^**^ |
| Social sensitivity | .39^***^ | .34^***^ | .22^**^ | .28^**^ | .19^*^ | .12 |
| Inequality of turn taking | .08 | .06 | .08 | .13 | .09 | .11 |
| WM accuracy | - | .24^*^ | .10 | - | .36^***^ | .23^**^ |
| Agreeableness | - | -.01 | .05 | - | .14 | .17^*^ |
| Conscientiousness | - | -.00 | -.04 | - | .08 | -.01 |
| Extraversion | - | -.21^*^ | -.10 | - | -.02 | .06 |
| Intellect | - | .09 | -.09 | - | -.03 | -.22^**^ |
| Neuroticism | - | .09 | -.02 | - | .06 | .01 |
| Intelligence | - | - | .32^**^ | - | - | -.03 |
| Confidence | - | - | .33^***^ | - | - | .63^***^ |
|  |  |  |  |  |  |  |
| R | .47 | .59 | .77 | .49 | .61 | .80 |
| R^2^ | .22 | .35 | .59 | .24 | .37 | .64 |
| ΔR^2^ | .22^***^ | .13^*^ | .24^***^ | .24^***^ | .13^*^ | .27^***^ |

*Note*. WM = Working Memory. β = standardised regression coefficient.

^***^ *p* < .001, ^**^ *p* < .01, ^*^ *p* < .05.

**Appendix E**

**Summary of LPA Goodness of Fit Indices and Model Selection**

LPA was performed for solutions with 2-6 classes (with 1 class as the default, see Table E1 on 6 predictor variables. These variables were individual intelligence and confidence, the extracted factors for CI and collective confidence, and individual and collective bias scores. Goodness of fit statistics were used to identify the number of latent classes (Henson et al., 2007; Marsh et al., 2009). Assessment of the indices and examination of the profiles within each model suggested a 3-Class solution was the best fitting model.

**Table E1**

*Goodness of Fit Statistics for All Latent Profile Analysis Models Tested (N = 105)*

| Classes in the model | AIC | Adjusted BIC | BIC | Entropy | BLRT | LogLik |  |
| --- | --- | --- | --- | --- | --- | --- | --- |
| 1 | 1806 | 1800 | 1838 | 1.00 | - | -891 |  |
| 2 | 1603 | 1590 | 1669 | 0.94 | 229^*^ | -776^***^ |  |
| 3 | 1560 | 1541 | 1661 | 0.96 | 69^*^ | -742^***^ |  |
| 4 | 1560 | 1534 | 1695 | 0.96 | 26 | -729^***^ |  |
| 5 | 1513 | 1480 | 1682 | 0.92 | 73^*^ | -692^***^ |  |
| 6 | 1490 | 1451 | 1694 | 0.92 | 49^*^ | -668^***^ |  |
| Class | Class counts and proportions for the latent classes | | Average latent class probabilities for most likely latent class membership (row) by latent class (column) | | |  |  |
|  | Counts | Proportions | Class 1 | Class 2 |  |  |  |
| Class 1 | 64 | .61 | .99 | .01 |  |  |  |
| Class 2 | 41 | .39 | .02 | .98 |  |  |  |
|  | Counts | Proportions | Class 1 | Class 2 | Class 3 |  |  |
| Class 1 | 57 | .54 | .99 | .00 | .01 |  |  |
| Class 2 | 15 | .14 | .00 | .94 | .06 |  |  |
| Class 3 | 33 | .31 | .05 | .00 | .95 |  |  |
|  | Counts | Proportions | Class 1 | Class 2 | Class 3 | Class 4 |  |
| Class 1 | 52 | .50 | .95 | .00 | .00 | .05 |  |
| Class 2 | 15 | .14 | .00 | .97 | .00 | .03 |  |
| Class 3 | 5 | .05 | .00 | .00 | 1.00 | .00 |  |
| Class 4 | 33 | .31 | .03 | .01 | .00 | .96 |  |

**Note.** AIC = Akaike Information Criterion; BIC = Bayesian Information Criterion; BLRT = Bootstrap Likelihood Ratio Test; LogLik = Log Likelihood of the data, given the model. *p*-values of the chi-squared test between k and k-1 solutions.

* *p* < .05; *** *p* < .001.

In particular, Akaike Information Criterion (AIC) values declined from the 1-Class to the 3-Class solution and then plateaued for the 4-Class solution. The Bootstrap Likelihood Ratio Test (BLRT) also suggested a 3-Class solution as it was a significant improvement on the 2-Class solution, but the 4-Class solution was not a significant improvement on the 3-Class solution. All solutions had acceptable entropy values - entropy greater than .80 is considered high (Clark & Muthén, 2009) – and entropy was greatest for the 3- and 4-Class solutions (.96). Entropy was reverse coded so 1 = complete certainty of classification and 0 = complete uncertainty. The sample adjusted Bayesian Information Criterion (BIC) and Log-likelihood of the data, given the model (LogLik) indicated that each new model improved the fit – suggested by decreasing values for each subsequent solution and *p*-values for less than .05.

The 2-, 3-, and 4-Class solutions all appear to be viable candidates for selection, so we compared them on the proportion of class membership, the goodness of classification, and the interpretability of each class. The 2- and 3-Class models had an adequate proportion of members (> .10) in each profile, but the 4-Class model did not (Class 3 = .05). Next, we checked the goodness of classification by examining the average latent class probabilities for the most likely latent class membership. All were 90% or greater for the assigned class which is acceptable (See Table C1: high values on the diagonal and low values off the diagonal indicated goodness of classification).

To select a model from the 2- and 3-Class solutions we examined the interpretations of the profiles within each model (see Figure E1). Both models contained low and high CI profiles, but the 3-Class solution also included an amplified CI profile whose members had significantly greater CI than individual intelligence. This third profile identified a small number of participants that benefitted the most from working together in a dyad and provided additional insight into systematic outcomes for individuals paired to work together. We conducted the same analyses (described below) on both the 2- and 3-Class solutions and the pattern of results was consistent. Thus, we selected the 3-Class solution as the best fitting model because it provided additional information beyond the 2-Class solution. All subsequent analyses reported in the main text are based on this model.

**Figure E1**

*Latent profile groups for 2- (A) and 3-Class (B) solutions. Error bars represent the standard error of the mean for each profile on each variable*

**
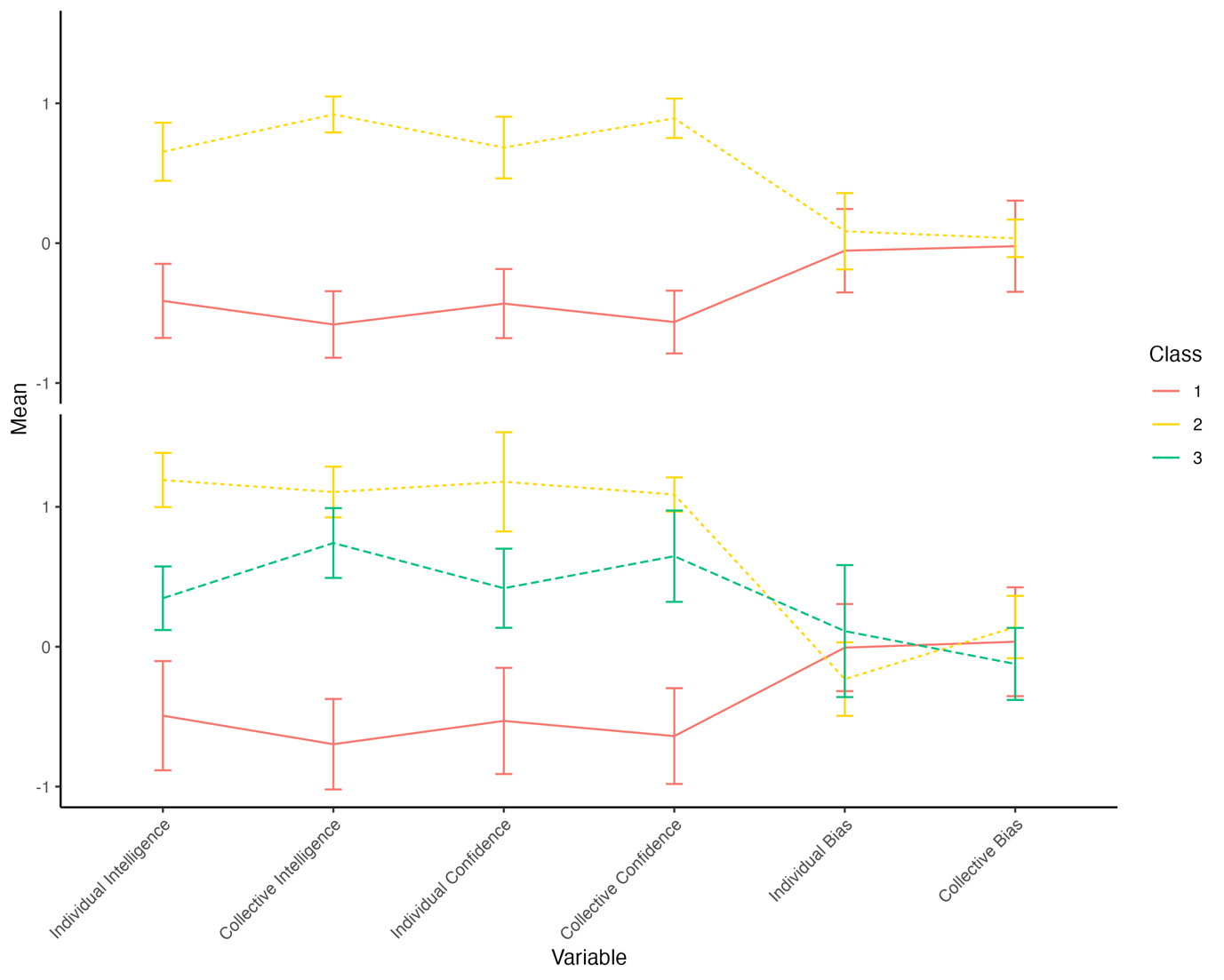
**
